# Female Chimpanzees Use Copulation Calls Flexibly to Prevent Social Competition

**DOI:** 10.1371/journal.pone.0002431

**Published:** 2008-06-18

**Authors:** Simon W. Townsend, Tobias Deschner, Klaus Zuberbühler

**Affiliations:** 1 School of Psychology, University of St Andrews, St Andrews, Scotland, United Kingdom; 2 Budongo Conservation Field Station, Masindi, Uganda; 3 Max Planck Institute for Evolutionary Anthropology, Leipzig, Germany; University of Sussex, United Kingdom

## Abstract

The adaptive function of copulation calls in female primates has been debated for years. One influential idea is that copulation calls are a sexually selected trait, which enables females to advertise their receptive state to males. Male-male competition ensues and females benefit by getting better mating partners and higher quality offspring. We analysed the copulation calling behaviour of wild female chimpanzees (Pan troglodytes schweinfurthii) at Budongo Forest, Uganda, but found no support for the male-male competition hypothesis. Hormone analysis showed that the calling behaviour of copulating females was unrelated to their fertile period and likelihood of conception. Instead, females called significantly more while with high-ranking males, but suppressed their calls if high-ranking females were nearby. Copulation calling may therefore be one potential strategy employed by female chimpanzees to advertise receptivity to high-ranked males, confuse paternity and secure future support from these socially important individuals. Competition between females can be dangerously high in wild chimpanzees, and our results indicate that females use their copulation calls strategically to minimise the risks associated with such competition.

## Introduction

In various animal species copulations are accompanied by a distinct vocal behaviour, the copulation call (e.g. African elephants (Loxodonta africana) [1], lions (Panthera leo) [2], elephant seals (Mirounga angustirostris) [3], and humans (Homo sapiens) [4]). Due to their prevalence, considerable debate has surrounded the adaptive significance of these conspicuous acoustic signals. In primates, copulation calls are loud, acoustically distinctive vocalisations emitted prior to, during or just after copulation. Calls can be produced by both males and females participating in the copulation, however in Old World monkeys and apes, it is more commonly females that vocalise [5]–[7]. Interestingly, not all copulations are accompanied by calling behaviour, suggesting that females have some control over call production.

A number of different hypotheses have been put forward to explain the adaptive significance of copulation calls [7], although it is unlikely that any one hypothesis in isolation is sufficient to explain call evolution. Indeed, copulation calls may operate at more than one level with multiple functions [8]. The most common hypothesis invoked to account for the evolution of such calls is that they are sexually selected traits to alert males, other than the mating partner, to the receptive condition of the female caller [3], [5], [6], [8]–[11], with the result of inciting competition amongst them. The incitation of male-male competition hypothesis [3] can operate at two distinct levels, which are not mutually exclusive [12]. Firstly, calls may operate to stimulate overt competitive interactions between males so that, indirectly, the female ends up with the most dominant partner [13]. Copulations accompanied by a call are predicted to primarily occur with low-ranking, less desirable males and increase subsequent levels of male aggression. Aggressive interactions can also occur during or after copulation to prevent insemination or future matings [9]. Secondly, copulation calls may lead to multiple mating partners, and this could generate additional benefits for the female due to sperm competition [10]. Under this scenario, males do not attempt to prevent insemination per se, but they should be particularly motivated to mate with the female shortly after a successful mating by another male. If female calling behaviour has been shaped by sperm competition, females should call to advertise ejaculation [10] and calling should decrease the interval between successive matings [8].

Polyandrous mating, and sperm competition that follows from it, increases paternity confusion for individual males, and it has been argued that this lowers the risk of male infanticide [10]. In contrast to the male-male competition hypothesis, however, the paternity confusion hypothesis makes no predictions about females trying to increase the quality of partners or sperm. Instead, females are primarily interested in receiving copulations from as many socially important partners as possible, safeguarding them from their infanticidal tendencies and gaining their future support. In many primate species females are notoriously vulnerable to infanticide [14], [15], suggesting that there are strong selective pressures acting on females to evolve behavioural or sexual counter-strategies to protect their infants: copulation calls may well be one such counterstrategy.

Although the theoretical reasoning behind the incitement of male-male competition and the paternity-confusion hypotheses is sound, the desired empirical support is weak, especially for chimpanzees. Most empirical work so far has been done with different monkey species, which are typically matrilineally bonded [8], [10], [13], [16]–[18], in contrast to male-bonded chimpanzees. A second relevant point is that if copulation calls function to increase a female's reproductive success, or confuse paternity amongst multiple males, then it is reasonable to predict that callers should take into account (a) at which stage in their cycle they are (b) whether the desired mating partners are present in the audience. A number of studies have investigated the influence of the female reproductive stage on vocal production. For example female-alpine accentors, Prunella collaris, sing only during their fertile time-period [19] and the stereotyped 50kHz vocalisations produced by female brown rats are only given during pro-oestrus [20]. In primates, it has also been suggested that copulation calls change based on female sexual status [10], [12], but hormonal data are not usually available to determine the precise time of ovulation.

Very little is known about the degree to which female primates adjust calling behaviour in relation to the composition of the audience. A growing body of evidence suggests that female-female competition, and the aggression that accompanies it, is far more pervasive in chimpanzee societies than previously thought [21]. Females are likely to compete with each other over access to resources and in mating systems where promiscuity is high, males and their sperm may be one such limiting resource [22]. For lower ranking, less competitively able females, it may thus not be beneficial to advertise successful matings with copulation calls if other females are nearby, especially if this increases the likelihood of aggression. Our pilot observations revealed that females often remained silent during copulations, although the reasons for this behaviour remained largely unknown [9]. Based on these considerations, we hypothesised that females adjusted their copulation calls, to maximise paternity confusion by soliciting copulations from nearby males on the one hand, and to minimise the effects of social competition caused by other females on the other hand. To address these points, we conducted a study on the copulation calling behaviour of wild female chimpanzees from the Sonso community of the Budongo Forest, Uganda.

## Results

### Mating behaviour of female chimpanzees

**Figure 1:**
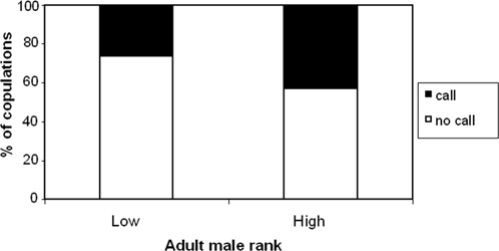
Copulation calls and the effect of male rank. Bar graphs showing the percentage of copulations accompanied by calls, N = 75, given by seven females when copulating with high (N = 5) and low (N = 3) ranking males.

**Figure 2:**
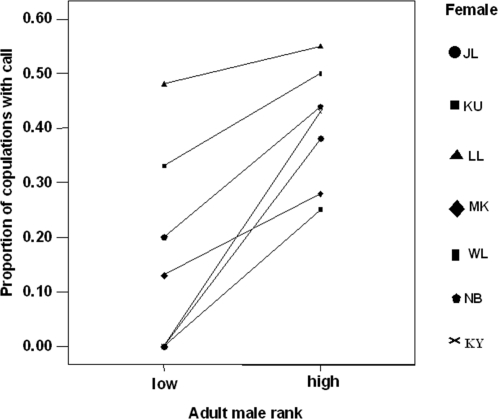
Individual variation in copulation calling behaviour. Line graphs showing the proportion of copulations accompanied by a call when copulating with high (N = 5) and low (N = 3) ranking males for each of the seven females.

All seven monitored females gave copulation calls during mating, but only in a minority of cases: The females copulated a total of 287 times and produced copulation calls during only 104 (36%) of copulations (table 1). The females were more likely to produce copulation calls when they mated with high-ranking adult males than low-ranking males (Wilcoxon exact test N _females_ = 7, Z = −2.37, p = 0.016, fig 1), with all seven females showing the same pattern (Cronbach's alpha test for reliability = 0.791, fig 2). There was no difference in calling behaviour when females copulated with low-ranked adult males and even lower-ranking subadult males (Wilcoxon exact test N _females_ = 7, Z = −0.405, p = 0.813)

35 (12%) observed copulations elicited aggression by a third party individual, either leading to interruption of the copulation or to targeted aggression to one of the mating partners within 10 min. There was no difference in the occurrence of aggression after silent or vocal matings (controlled for copulation number: N _silent_ = 26, N _vocal_ = 9, binomial test (0.63), p = 0.2 2-tailed). Out of the nine instances of aggression following a vocalisation, four were caused by high-ranking females, three by high-ranking males, and two by low ranking males. These four cases of female-caused aggression were particularly severe and always directed at a low-ranking female. If the same four low-ranking females copulated, but remained silent, then the high-ranking females never responded with aggression. In no case were they likely to see the copulation event (Fishers exact test, 2-tailed, N_silent_ = 4, N_vocal_ = 4, p = 0.02).

**Table 1:** Rank and copulation calling behaviour of seven adult females of the Sonso community, Budongo Forest, Uganda

Female	Female rank	Total number of copulations	% copulations accompanied by a call
**LL**	Low	66	53
**WL**	Low	68	34
**NB**	Alpha	50	38
**MK**	Low	37	35
**KU**	Low	18	11
**KY**	High	29	24
**JL**	Mid	19	26
**Total**		287	36

Finally, we found no relation between the time interval between successive copulations with different males and the likelihood of a female producing copulation calls (Wilcoxon exact test; Z = −0.314, N_silent_ = 6, N_vocal_ = 6, p = 0.844).

### Hormonal analyses

We were able to analyse the hormonal profiles of six complete oestrus cycles (LL: N = 1; WL: N = 3, NB: N = 2), which allowed us to determine the exact time of ovulation. Females called prior to the fertile peri-ovulatory period (Pre-POP), during the fertile peri-ovulatory period (POP) and after ovulation (Post-POP). Because one female (WL) did not exhibit a Post-POP period and another (NB) did not exhibit a Pre-POP period, only five cycles were included for each analysis. We found no significant difference in the calling rate between Pre-POP and POP periods (Binomial GLMM with female ID as a random factor Z = −0.789, N = 121, p = 0.430) or between POP and Post-POP (Binomial GLMM with female ID as a random factor, Z = −1.344, N = 117, p = 0.181)

### Audience effects

**Figure 3:**
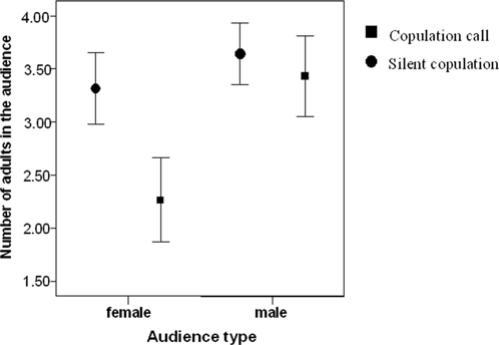
Audience effects. Mean number of individuals in the audience in the presence/absence of a copulation call. Female audience (N_females_ = 5, N_copulations_ = 90): the number of adult females present. Male audience (N_females_ = 7, N_copulations_ = 126): the number of adult males present. Error bars represent Mean+−1 SE.

To test for audience effects we randomly selected for each of the seven females an equal number of copulations (N = 18), which were subjected to analyses, i.e. N = 126 total. Adult male audience size had no effect on call production by the copulating female (Wilcoxon Exact test Z = −1.10, N_females_ = 7, p = 0.328; fig 3), despite the fact that there were consistently more high-ranking males present when a female copulated with a high-ranked male (Paired T test; t = −4.916, N_females_ = 7, p <0.001). In contrast, the number of adult females in the party had a significant effect on call production (Mann Whitney U test: U = 536, N_silent_ = 62, N_vocal_ = 28. N_females_ = 5, p = 0.04, fig 3); females called less the more adult females were in the party. Sample sizes were too small for two females (NB, KY), who were excluded from this analysis. Both were high-ranking females and there were indications that they behaved differently in the presence of other females, compared to the other five lower-ranking females. The observed audience effect was mainly driven by the social position of listening females. Females called significantly less if they were surrounded by a female audience that contained individuals of equal or higher rank than themselves (Mann Whitney U test: U = 516, N_silent_ = 62, N_vocal_ = 28, N_females_ = 5, p = 0.025).

**Figure 4:**
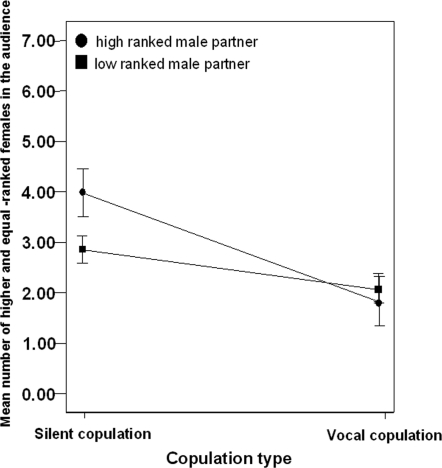
Male rank and female audience composition interaction. Line graphs showing the mean number of high and equal ranked females in the audience when copulating with a) high ranked males and b) low ranked males. Error bars represent Mean+−1SE.

We were particularly interested in how female audience composition affected calling behaviour. To identify the independent and potentially interactive influence of the determining variables we conducted a binary logistic regression. Of the variables tested male rank, female audience composition and male rank*female audience composition explained a significant proportion of the overall variance (binary logistic regression with female ID as a random factor χ^2^ = 8.595, Nmale rank p = 0.004, female audience composition p  = 90, Nagelkerke r^2^ = 0.421, equals; 0.029, male rank*female audience composition p = 0.043). The model explained variation in female calling behaviour with 82% accuracy, a rate significantly higher then that when running the model with no explanatory variables (Binomial (0.7) p = 0.024 2-tailed). The significant interaction effect suggested that the females' response to female audience composition also depended on the rank of the male mating partner. Whilst there was a trend to call less when more high-ranking females were in the audience for both rank groups, this was most apparent when females copulated with high-ranked males (fig 4).

## Discussion

Overall, our study lent no support to the ‘male-male competition’ hypothesis of copulation calling [3], despite its prominence in the sexual behaviour literature. Specifically, females did not produce calls when mating with low-ranked males in order to instigate disruption by high-ranked individuals [7], [18]. Instead, they called more when mating with higher-ranked males, an effect also reported in other primate species [10]–[12], [18]. In our sample, copulation calls did not lead to increased levels of aggression towards the mating pair. We also found no evidence that chimpanzee copulation calls operated at the ‘sperm competition’ level [10]. Whilst it was virtually impossible to determine the occurrence of ejaculation, the duration to the next copulation was unrelated to the female's calling behaviour. Instead, females produced copulation calls preferably when mating with high-ranked adult males, but suppressed calls if high-ranked females were present. Hormonal analysis showed that female calling behaviour was unrelated to their fertile period and likelihood of conception.

If the male-male competition hypothesis does not explain copulation calling behaviour, then why do females call? Our study suggests that social variables are important in driving these vocalisations. Females call significantly more when copulating with high- compared to low-ranked partners, and since other dominant males are usually nearby in these circumstances, calling is one potential strategy allowing a female to signal her receptivity to a large audience of high-ranked males. Although females appear to be motivated to advertise their receptivity, they do not provide any information about the timing of their ovulation, a pattern that also holds for Barbary macaques where precise information on the timing of ovulation is not available in copulation calls [22] [but see also 23]. By calling in the presence of high-ranking adult males and by concealing ovulation, females may prevent monopolisation by a single male and avoid decreased paternity certainty by other males.

Research from long-term field studies increasingly shows that chimpanzee females are exposed to severe social pressure from other group members, especially when resources are limited. Our own research has shown that female chimpanzees can suffer substantially from infanticide-related threats [21]. In this context, confusing paternity, particularly amongst socially important males, has a two-fold advantage. Firstly, it reduces the probability that males will attack infants potentially sired by them [10]. Secondly, it is likely to improve a male's general willingness to provide support, including during female-initiated agonistic encounters. Possibly because of their previous mating history, high-ranking males have been observed to intervene during female aggressive events, which in some cases have resulted in female-led infanticidal attacks, at Gombe [24], [25], Mahale [26] and Budongo [21]. Our data are consistent with the idea that chimpanzee females may use copulation calls to minimise these threats. Other fission-fusion species (lions [27] and hyenas (Crocuta crocuta) [28]) at risk to infanticide display behavioural counter-strategies such as avoiding the group around parturition. Whilst female chimpanzees have been observed to employ similar behaviours, they may also use copulation calls–a vocal counter-strategy- to manage their risks.

Chimpanzees produce copulation calls at much lower rate than other primates [7], suggesting females take other factors into account, apart from trying to increase paternity confusion. Our data suggest that lower ranking females refrained from calling when mating with high-ranking males if high-ranking females were nearby, suggesting that they were trying to conceal their sexual activity in these circumstances.

Unlike most other primates, chimpanzee females leave their natal group at adolescence to immigrate into neighbouring communities. Immigration will affect the adult sex ratio of a group [21], increasing competition for resources between females, such as high-quality foraging areas [29], [30], and possibly the amount and quality of available sperm [31]–[37]. As a consequence, more competitively able high-ranking females should have an interest in maximising their own access to such resources and escalated aggression may be one strategy [21], [25], [29], [30]. One counterstrategy for lower-ranked females is to form short-term associations with the adult males of the community [Kahlenberg personnel communication] and, as suggested by this study, to modify their copulation calling behaviour [38] when high-ranking resident females are likely to witness their sexual activities. Copulation calls may therefore act as a flexible sexual strategy against the risk posed by other females within a chimpanzee community.

To conclude, female copulation calls in primates and other groups of animals have usually been interpreted as male-directed signals, for example to advertise fertility and incite male-male competition, but our findings in wild chimpanzees do not support this view. In our study, chimpanzee females adjusted their calling behaviour in flexible ways, potentially to avoid aggression from other females and possibly to secure future benefits from the socially important males. Data from more females and different study sites will be required to test this hypothesis more thoroughly. For many years, female chimpanzees have been regarded as the more peaceful sex. However, there is increasing evidence from a number of communities studied in the wild, which indicates that female competition plays an important role in dictating female behaviour and our data provide further support for this view. Our study indicates that the social pressures deriving from resource competition have acted as an important selective force, shaping the copulation calling behaviour in wild chimpanzees.

## Materials and Methods

### Study site and animals

We studied the Sonso community of the Budongo Forest, Uganda [39], during two field seasons (January 2006-April 2006 and October 2006-March 2007). The community has been habituated since 1991 and provisioning has never been used. During the period of study the group comprised 78 individuals including 8 adult males and 25 adult females. Of the 25 adult females, data were collected from 7 adult females. Three additional females also had sexual swellings and copulated during the study but were excluded from analyses due to low copulation frequency (<15 copulations).

### Copulation calls, behaviour and determination of female swelling size

**Figure 5:**
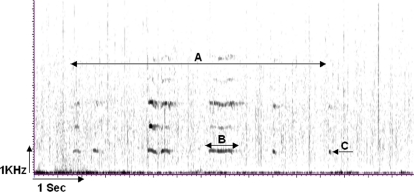
Copulation call spectrogram. Time-frequency spectrogram of a female copulation call from Budongo Forest during maximum tumescence. Filter bandwidth: 159 Hz, Frequency resolution: 86.1 Hz. Depicted is (A) the total copulation calling bout of approximately 6.5 s and (B) a single copulation call of approximately 0.6 s by the female JL. (C) The lowest visible band is the fundamental frequency from which acoustic measurements were taken with three visible harmonic bands. Copulation calls have a frequency range of 700–1000 Hz.

Around the time of ovulation, female chimpanzees exhibit sexual swellings. The average duration of the maximum swelling period is about ten days [40]–[42] and females almost exclusively copulate during this period [43]. Females mate promiscuously with multiple males [40], [43], but they do not produce a vocalisation every time [9]. Copulation calls consist of a rhythmic succession of high-frequency squeaks or screams and typically begin during the copulation, after mounting and intromission (fig 5). Copulation calls can be reliably identified by human observers and are audible in forest habitats up to about 50 m.

Copulations from cycling adult females were collected using all day focal follows on each day of the female's maximum tumescence phase. Given that only one female could be followed for this duration, yet more than one female could cycle at any one time, ad-libitum observations of copulations were also taken. Only copulations occurring during the maximum tumescence phase were considered. Maximum tumescence was determined following Furuichi's [44] method, which uses degree of wrinkling of the sexual swelling (on a 4 point scale at Budongo) as the main parameter, rather than labial occlusion [45]. Sexual skin swelling characteristics were recorded every morning through visual inspection of the perineal area. Inter-observer agreement between ST and his field assistant Monday Gideon (MG) was a pre-requisite for final assessment of female swelling size. In addition to swelling size, we noted the following variables: identity of mating partners, presence/absence of copulation call, temporal occurrence of call in relation to copulation, aggressive behaviours following a copulation, duration to next copulation and composition of the audience during copulation. Only calls that occurred during the copulation were considered to control for the vocalisation being elicited by an alternative stimulus other than the copulation.

### Urine sample collection, hormone analysis, and assessment of the fertile period

**Figure 6:**
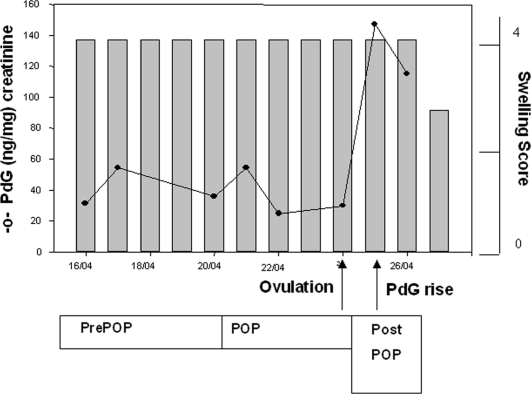
Chimpanzee ovulation profile. Profiles of urinary pregnandiol in ng/mg creatinine and perineal swelling for the adult female WL during April 2007. POP: periovulatory or fertile period, defined as the day of ovulation plus the three preceding days; PrePOP: period of maximum tumescence prior to POP PostPOP: period of maximum tumescence following POP

To determine approximate timing of ovulation, we collected regular urine samples during the period of maximum tumescence, with sampling gaps of no greater than two days. Samples were collected directly after an individual had been observed urinating by aspiration of the urine from plastic sheets or vegetation using disposable plastic pipettes. They were stored in 2-ml polypropylene Cryotubes in liquid nitrogen until shipment on dry ice to the laboratory. Samples were analysed for immunoreactive pregnanediol glucuronide (PdG), using enzyme immunoassay procedures [46]. The sensitivity of the assay at 90% binding was 12.5 pg. Serial dilutions of urine samples of the follicular and luteal phase gave displacement curves parallel to those obtained with the appropriate standard. Intra- and inter-assay coefficients of variation, calculated from replicate determinations of quality controls were 7.94 and 6.52% (high) and 13.31 and 11.26% (low) respectively. To compensate for variations in the volume and concentration of urine samples, all hormone levels were divided by the urinary creatinine concentration as described in Bahr et al. [47]. Based on the defined postovulatory rise in PdG levels, the day of ovulation was presumed as the day preceding the day of PdG increase ([42], [48], fig 6). Based on human data regarding the survival time of ovum and sperm, the fertile period (POP) was defined as the day of ovulation plus the three preceding days [42], with the post-ovulation period being the period of maximum tumescence following POP.

### Behavioural observations

#### Male-male aggression

We scored all instances of aggression during copulations, and during the subsequent 10-minute time-window, provided we could identify a target of aggression. Aggressive events could range from ‘mild’, such as arm raises or displays, to ‘severe’, such as chases or stamping and beatings [49].

#### Sperm competition

In wild chimpanzees it is difficult to determine reliably whether or not ejaculation has occurred [50]. Sperm competition has alternatively been assessed indirectly, by measuring the time interval between successive copulation events [8]. The prediction is that sperm competition increases as the time interval decreases.

#### Male rank

In chimpanzees dominance rank is usually assessed by using the occurrence and direction of pant-grunt vocalisations. The direction of these vocalisations is regarded as a good indicator of relative social status [38], [51]–[53]. Because of the instability of the male hierarchy at the time of study, it made little sense to attempt to construct a linear dominance hierarchy. Instead we determined the status to each male by calculating the proportion of other males in the community from whom he received pant grunt vocalisations, allowing us to assign each individual with a ‘dominance value’ (DV = arcsine of the square root of the proportion; [54]). Eight of the community adult males received pant-grunts from other males. There were two clusters of individuals with similar DV scores; 5 high-ranking males (NK, DN, ZF, BB, MA) and 3 low ranking males (GS, MS, BO). Juvenile and sub-adult males were not observed to receive any pant-grunts.

#### Audience effects

Wild chimpanzees adjust call production depending on who is likely to listen to their calls [49]. They usually travel in small family groups, consisting of a mother and her dependent offspring, or in mixed-sex parties of different sizes, usually around 10 individuals. Party composition is relatively fluid, with individuals joining or leaving regularly, and group members are often not in direct visual contact. To determine whether the audience had an impact on copulation calling we noted party composition at 15-minute intervals when following a female. A party was defined as any individual within a 50 m radius [39] of the focal female. Every time a copulation event occurred, we (ST, MG) conducted an additional and more detailed search of the area to account for individuals that might have joined or left the party since the previous scan. This was particularly important for copulations that occurred in trees, where the female has a better observational vantage point than observers on the ground.

#### Female rank

Female rank was determined in a previous study of female-male aggression [55]. Rank relations between female chimpanzees are more stable than between males [56], and there was no evidence of any significant changes since that study.

### Statistical analyses

Whenever possible we conducted parametric analyses. If the data failed to meet conditions for parametric analyses, before and after transformation, we used non-parametric statistics. A binary logistic regression was used to identify the influence of the following independent variables on copulation calling: female audience composition, male rank, and male audience number [57]. All tests were two-tailed and significance levels were set at α = 0.05. For small sample sizes, we calculated exact p-values, as recommended by Mundry and Fischer [58]. All described statistical analyses were done using SPSS v. 15.0 and R version 2.5.1 (R Core Development Team, 2007)

## References

[1] Poole JH, Payne KB, Langbauer W, Moss CJ (1988). The social contexts of some very low frequency calls of African elephants.. Behav Ecol Sociobiol.

[2] Schaller GB (1972). The Serengeti lion: a study of predator–prey relations..

[3] Cox CR, LeBoeuf BJ (1977). Female incitation of male competition: a mechanism in sexual selection.. Am Nat.

[4] Hamilton WJI, Arrowood PC (1978). Copulatory vocalizations of chacma baboons (Papio ursinus), gibbons (Hylobates hoolock), and humans.. Science.

[5] Hauser MD (1996). The evolution of communication..

[6] Semple S (2001). Individuality and male discrimination of female copulation calls in the yellow baboon.. Anim Behav.

[7] Pradhan G, Engelhard A, van Schaik CP, Maestripieri D (2006). The evolution of female copulation calls in primates: a review and a new model.. Behav. Ecol. Sociobiol.

[8] Semple S (1998b). The function of Barbary macaque copulation calls.. Proc R Soc Lond B.

[9] Hauser MD (1990). Do Chimpanzee copulatory calls incite male-male Competition?. Anim Behav.

[10] O'Connell SM, Cowlishaw G (1994). Infanticide avoidance, sperm competition and mate choice: the function of copulation calls in female baboons.. Anim Behav.

[11] Oda R, Masataka N (1995). Function of copulatory vocalizations in mate choice by female Japanese macaques (Macaca fuscata).. Folia Primatol.

[12] Semple S, McComb K, Alberts S, Altmann J (2002). Informational content of female copulation calls in yellow baboons.. Am J Primatol.

[13] Henzi SP (1996). Copulation calls and paternity in chacma baboons.. Anim Behav.

[14] van Schaik CP, van Schaik CP, Janson CH (2000). Infanticide by male primates: the sexual selection hypothesis revisited.. Infanticide by males and its implications.

[15] Muller MN, Kahlenberg S, Emery Thompson M, Wrangham RW (2007). Male coercion and the costs of promiscuous mating for female chimpanzees.. Proc R Soc Lond B.

[16] Hauser MD (1993). Rhesus monkey (*Macaca mulatta* ) copulation calls: Honest signals for female choice?. Proc R Soc Lond B.

[17] Hauser M (2007). When males call, females listen: sex differences in responsiveness to rhesus monkey *Macca mulatta*, copulation calls.. Anim Behav.

[18] Nikitopoulos E, Arnhem E, van Hooff J, Sterck E (2004). Influence of female copulation calls on male sexual behavior in captive Macaca fascicularis.. Int J Primatol.

[19] Langmore NE, Davies NB, Hatchwell BJ, Hartley IR (1996). Female song attracts males in the alpine accentor *Prunella collaris*.. Proc R Soc Lond B.

[20] Matochik JA, White NR, Bar¢eld RJ (1992). Variations in scent marking and ultrasonic vocalizations by Long-Evans rats across the estrous cycle.. Physiol Behav.

[21] Townsend SW, Slocombe KE, Emery-Thompson M, Zuberbühler K (2007). Female-led infanticide in wild chimpanzees.. Curr Biol.

[22] Pfefferle D, Brauch K, Heistermann M, Hodges JK, Fischer J (2008). Female Barbary macaque (Macaca sylvanus) copulation calls do not reveal the fertile phase but influence mating outcome.. Proc R Soc Lond B.

[23] Semple S, McComb K (2000). Perception of female reproductive state from vocal cues in a mammal species.. Proc R Soc Lond B.

[24] Pusey AE (1980). Inbreeding avoidance in chimpanzees.. Anim Behav.

[25] Pusey AE, Murray CM, Wallauer W, Wilson ML, Wroblewski E Severe aggression among female chimpanzees at Gombe National Park, Tanzania.. Int J Primatol (In press).

[26] Nishida T, Heltne PG, Marquardt LA (1989). Social interactions between resident and immigrant female chimpanzees.. Understanding Chimpanzees.

[27] Packer C, Pusey AE, Eberly LE (2001). Egalitarianism in female African lions. Science.

[28] East ML, Hofer H, Turk A (1989). Functions of birth dens in spotted hyaenas (Crocuta crocuta).. J Zool Lond.

[29] Kahlenberg SM, Emery Thompson M, Wrangham RW Female competition over core areas among Kanyawara chimpanzees, Kibale National Park, Uganda.. Int J Primatol (in press).

[30] Pusey AE, Williams J, Goodall J (1997). The influence of dominance rank on the reproductive success of female chimpanzees.. Science.

[31] Nakatsura K, Kramer DL (1982). Is sperm cheap? Limited male fertility and female choice in the lemon tetra (Pisces, Characidae).. Science.

[32] Dewsbury DA (1982). Ejaculate cost and male choice.. Am Nat.

[33] Van Voorhies WA (1992). Production of sperm reduces nematode life-span. Nature.

[34] Gage MJG, Cook PA (1994). Sperm size or number? Effects of nutritional stress upon eupyrene and apyrene sperm production strategies in the moth Plodia interpunctella (Lepidoptera: Pyralidae).. Funct Ecol.

[35] Preston BT, Stevenson IR, Pemberton JM, Wilson K (2001). Dominant rams lose out by sperm depletion.. Nature.

[36] Weddell N, Gage MJG, Parker GA (2002). Sperm competition, male prudence and sperm-limited females. Trends Ecol.. Evol.

[37] Marson J, Gervais D, Meuris S, Cooper RW, Jouannet P (1989). Influence of ejaculation frequency on semen characteristics in chimpanzees (Pan troglodytes).. J Reprod Fert.

[38] De Waal F (1982). Chimpanzee Politics. Jonathan Cape, London.

[39] Reynolds V (2005). The chimpanzees of the Budongo forest-Ecology, behaviour, and conservation..

[40] Tutin CEG (1979). Mating patterns and Reproductive strategies in a community of wild chimpanzees (Pan troglodytes schweinfurthii).. Behav Ecol Sociobiol.

[41] Hasegawa T, Hiraiwa-Hasegawa M (1983). Opportunistic and restrictive matings among wild chimpanzees in the Mahale mountains, Tanzania J Ethol.

[42] Deschner T, Heistermann M, Hodges K, Boesch C (2003). Timing and probability of ovulation in relation to sex skin swelling in wild West African chimpanzees, Pan troglodytes verus.. Anim Behav.

[43] Goodall J (1986). The chimpanzees of Gombe: patterns of behavior..

[44] Furuichi T (1987). Sexual swelling, receptivity, and grouping of wild pygmy chimpanzee females at Wamba, Zaire.. Primates.

[45] Dahl JF (1999). Perineal swelling during gestation and maternal competence in chimpanzees.. J of Med Primatol.

[46] Heistermann M, Möhle U, Vervaecke H, van Elsacker L, Hodges JK (1996). Application of urinary and fecal steroid measurements for monitoring ovarian function and pregnancy in the bonobo (*Pan paniscus*) and evaluation of perineal swelling patterns in relation to endocrine events.. Biol of Reprod.

[47] Bahr NI, Palme R, Möhle U, Hodges JK, Heistermann M (2000). Comparative aspects of the metabolism and excretion of cortisol in three individual nonhuman primates.. Gen and Compar Endocrin.

[48] Deschner T, Heistermann M, Hodges K, Boesch C (2004). Female sexual swelling size, timing of ovulation and male behaviour in wild West African chimpanzees.. Horm and Behav.

[49] Slocombe KE, Zuberbühler K (2007). Chimpanzees modify recruitment screams as a function of audience composition.. P Natl Acad Sci.

[50] O'Hara SJ, Lee PC (2006). High Frequency of Postcoital Penis Cleaning in Budongo Chimpanzees.. Folia Prim.

[51] Bygott JD, Hamburg DA, McCown ER (1979). Agonistic behaviour, dominance, and social structure in wild chimpanzees of the Gombe National Park.. The great apes.

[52] Boesch C, Boesch H (2000). The Chimpanzees of the Tai Forest: Behavioral Ecology and Evolution..

[53] Newton-Fisher NE (1997). Tactical behaviour and decision making in wild chimpanzees..

[54] Beilharz RG, Mylrea PJ (1963). Social position and behaviour of dairy heifers in yards.. Anim Behav.

[55] Newton-Fisher NE (2006). Female coalitions against male aggression in wild chimpanzees of the Budongo forest.. Int J Prim.

[56] Wittig R, Boesch C (2003). Food competition and linear dominance hierarchy among female Pan troglodytes verus of the Taï National Park.. Int J Prim.

[57] Wilson ML, Hauser MD, Wrangham RW (2005). Does participation in intergroup conflict depend on numerical assessment, range location or rank of wild chimpanzees.. Anim Behav.

[58] Mundry R, Fischer J (1998). Use of statistical programs for nonparametric tests of small samples often leads to incorrect P values: examples from *Animal Behaviour*.. Anim Behav.

